# Differences and similarities in the conceptualization of COVID-19 and other diseases in the first Italian lockdown

**DOI:** 10.1038/s41598-021-97805-3

**Published:** 2021-09-15

**Authors:** Claudia Mazzuca, Ilenia Falcinelli, Arthur-Henri Michalland, Luca Tummolini, Anna M. Borghi

**Affiliations:** 1 BallaB (Body, Action, Language Lab), Department of Dynamic and Clinical Psychology, and Health Studies, Sapienza University of Rome, Rome, Italy; 2 University of Montpellier-LIFAM, Montpellier, France; 3 Institute of Cognitive Sciences and Technologies, Italian National Research Council, Rome, Italy

**Keywords:** Psychology, Human behaviour

## Abstract

Several studies have highlighted the flexible character of our conceptual system. However, less is known about the construction of meaning and the impact of novel concepts on the structuring of our conceptual space. We addressed these questions by collecting free listing data from Italian participants on a newly–and yet nowadays critical–introduced concept, i.e., COVID-19, during the first Italian lockdown. We also collected data for other five illness-related concepts. Our results show that COVID-19’s representation is mostly couched in the emotional sphere, predominantly evoking *fear*—linked to both possible health-related concerns and social-emotional ones. In contrast with initial public debates we found that participants did not assimilate COVID-19 neither completely to severe illnesses (e.g., tumor) nor completely to mild illnesses (e.g., flu). Moreover, we also found that COVID-19 has shaped conceptual relations of other concepts in the illness domain, making certain features and associations more salient (e.g., flu-fear; disease-mask). Overall, our results show for the first time how a novel, real concept molds existing conceptual relations, testifying the malleability of our conceptual system.

## Introduction

What comes to our mind when we read the word “COVID-19”? Does the way in which we think of COVID-19 resemble how we think of other diseases, such as tumor and flu? And if so, to what extent?

The term COVID-19 has been coined by the World Health Organization (WHO) on February 11, 2020^1^ as an acronym for the COronaVIrus Disease 19, a respiratory syndrome caused by the new coronavirus SARS-CoV-2, first discovered in Wuhan, China, in December 2019. Initially, the fact that this new viral disease shared many symptoms with seasonal diseases (e.g., cold, flu) gave rise to a heated public debate. In this context, COVID-19 has been frequently assimilated to these more familiar diseases, thus creating a long-lasting confusion in the population. Understanding how COVID-19 is represented is therefore important for practical reasons: knowing how laypeople conceptually represent COVID-19 can support practitioners, scientists, and politicians to deal with invisible barriers that may facilitate the spread of this pandemic^2^. At the same time, it is important for scientific reasons, given that it is rare to observe the emergence of a new concept quickly spreading on a global scale, affecting people’s everyday life, and that might also impact the representation of well-established conceptual domains (e.g., diseases).

Other concepts, such as those related to technology (e.g., smart-phone, social network), have emerged and rapidly spread in recent years. However, previous work on novel concepts often concentrated on artificial categories created by the experimenters. For instance, Granito, Scorolli and Borghi^3^ presented participants with novel concrete and abstract categories formed by Lego bricks and asked them to freely categorize them after having received either a sensorimotor or a linguistic training. Afterwards, they performed a categorical recognition task. The results show that abstract categories were more difficult to form, and benefited more from the linguistic training compared to concrete categories, suggesting a critical role of sociality and language in the acquisition of more abstract entities. We chose to focus on COVID-19, aside from its intrinsic interest, not because it is representative of novel concepts, but because of its peculiar character. It is peculiar for at least two reasons: because it is easy to determine when it was introduced, and because no other concept has spread so quickly across different countries and populations. In addition, in Italy as in many other countries, it extensively attracted the media's attention. So, the first reason motivating our study was the desire to understand how people in the first wave of the pandemics represented COVID-19. Which kinds of conceptual relations did it evoke? Did people think more of its symptoms, of its social consequences, of the emotions it evokes? Did they represent it more like a severe disease, such as a tumor, or maybe like a disease easier to deal with, such as a flu? Accessing the general public’s conceptual representation of this new, complex, and yet extremely salient concept is therefore the first aim of this study.

The second reason to investigate COVID-19 representation has implications more directly relevant to the literature on concepts. Concepts can be considered aggregates of experiences in semantic memory, characterized by a certain degree of stability^4^. They connect our past with current and future experiences, gluing them together^5^. Furthermore, concepts are flexible entities, continuously updated in light of novel experiences. Indeed, many authors recognized the importance of investigating not only stable but also variable aspects of concepts (e.g.^6–8^). In the case of COVID-19, we have witnessed a new phenomenon: the emergence of a new concept, the use of which spread very rapidly. Initially known only in laboratories and in specialist settings, this concept rapidly entered our houses and became commonly used. Importantly, the concept of COVID-19 is continuously updated, discussed, and refined by an entire community of knowledge composed of scientists, practitioners, and politicians^9^. In addition, given the initial parallelisms that have been drawn between COVID-19 and other diseases, we might assist to a bidirectional influence on the semantic space of diseases. For instance, on the one hand the newly gained information on COVID-19 might impact previous knowledge related to other diseases, while on the other hand established knowledge of other diseases might serve as a scaffolding for reinforcing new conceptual associations needed for the concept of COVID-19. So, the second aim of this study is to assess whether–and to what extent–the introduction of COVID-19 has affected more established concepts, and what is the impact of these concepts on the conceptual representation of COVID-19.

Concepts are flexible entities, receptively adapting to new situations. Not only different extensive experiences (e.g., practicing sports, playing instruments) modulate our conceptual system (e.g.^10,11^), but even engaging in varied sensorimotor training for three weeks can impact objects’ conceptual representation (e.g.^12^). Conceptual activation is further affected by task conditions. For instance, the modality in which stimuli are presented to participants (e.g., auditory, visual) affects response times in property-verification tasks. Van Dantzig, Pecher, Zeelenberg and Barsalou^13^, for example, found slower response times for trials preceded by a perceptual trial presented in an incongruent modality with respect to those presented in congruent modalities. So, concepts can flexibly re-enact relevant information related to a given category depending on the specific situation (^14^ for reviews, see^15–17^).

Language is a further source of conceptual flexibility. Cross-linguistic and cross-cultural investigations demonstrated that conceptual representations are carved up by languages very differently across cultures^18^. Several domains including mental states, events, time, and spatial relations (see^19,20^), but also seemingly naturally-bounded entities such as body parts^21^ dramatically vary in their conceptual representation depending on the language investigated. In sum, language contributes to our reality’s co-construction in many different ways^22^. Among these, the information we gain from public discussions, experts, and media greatly contribute to shaping and refining our conceptual system^23–25^. This might be especially true for relatively new concepts, or concepts initially mainly used in specialist settings, such as “COVID-19”.

Remarkably, in the present study we are able to timely investigate how a real concept, recently introduced and often the object of public discussions, is represented by laypersons. This constitutes the first important novelty of our work: compared to previous studies addressing the consolidation of meanings, often focused on artificial categories, our study directly tackles the emergence and representation of a new concept. In addition, here, we sought to assess whether and how the introduction of a new concept further carved the conceptual relations of semantically related concepts. In fact, while studies dealing with conceptual flexibility provided evidence that task conditions, points of view, and cultural and linguistic environment shape conceptual representations, the impact of the introduction of a new concept on such a large-scale on the semantic space is clearly understudied. This unique opportunity to address conceptual flexibility represents the second focal novelty of our work.

### The current study: how is COVID-19 conceptualized, and what is its semantic relation with other disease concepts?

In the present study, our aim was to investigate how people conceptualize a relatively new concept, i.e., COVID-19, and whether and how the introduction of this concept shaped the semantic associations of other concepts in the disease domain. Importantly, we recruited participants from Italy, a country severely hit by COVID-19, and the data were collected in the first wave of the pandemic, during lockdown—the first national lockdown in the world. So, the timing of the data collection allowed us to capture the emerging meaning of COVID-19 in its initial phase.

To tackle these questions, here we apply a method typically used to investigate conceptual representations, i.e., a semantic fluency task. The main assumption underlying semantic fluency tasks is that when participants are presented with a target word, concepts that are semantically related to it will be immediately activated and produced. Unlike explicit questions concerning attitudes or definitions, semantic fluency tasks are thought to be an indirect measure of psychological proximity of concepts, hence providing access to semantic memory. This family of methods is widely employed in neuropsychology, to measure the semantic integrity of certain domains in patients with brain damage^26^, or to assess memory organization in schizophrenic individuals^27^. Anthropologists and linguists make a consistent use of semantic fluency tests too, to build folk taxonomies of how specific cultural domains are conceptualized by a particular cultural group^28^. Different varieties of semantic fluency tasks exist. Participants might be required to produce features that are typically true of a given concept (i.e., feature generation task; e.g.^20,29^), or they can be asked to produce all the words that come to their mind (i.e., free-listing task; e.g.^30–32^). Semantic property norms collected through these methods have been extensively employed^33^ to show that words with more semantic associates tended to be responded to faster and/or more accurately in various semantic tasks^34^. Here, we chose to use a free-listing task because we were dealing with a novel concept, and we were especially interested in the dynamic aspects that characterized the pattern of produced relations. We wanted to capture the whole pattern of elicited conceptual relations, avoiding constraining participants to produce only properties that are true of the concept. Importantly, free-listing tasks have also been used to understand how people represent concepts^35,36^. In free-listing tasks, concepts that are mentioned earlier and more frequently in a given list are thought to be more psychologically salient for the target concept. So, this method allows us to address how we represent the concept of COVID-19, and how and to what extent its representation is similar to that of five further semantically related concepts, i.e., disease, virus, flu, fever, tumor.

Furthermore, we were interested in understanding whether and to what extent the introduction of this new concept has changed an entire semantic field, i.e., modifying the relationship between more and less severe diseases, from “flu” to “tumor”. Finally, we intended to capture whether the associations to other similar concepts, i.e., diseases, have been restructured in light of the spread of COVID-19—for example, whether terms specifically related to COVID-19 appear also among the features listed with other concepts.

## Results

### Correlation between demographic information, perception of risk, and level of information

Before moving to the analysis of free-listing data, we addressed whether participants had a different perception of COVID-19 related risks (tested with a continuous scale from 1 to 7) depending on three main parameters: their regional provenience (North of Italy; Center of Italy; South of Italy), the frequency with which they received information and news about COVID-19 (never; rarely; sometimes; often; very often), and the number of cases positively tested for COVID-19 within regions at the time of participation—as reported by the National website https://github.com/pcm-dpc/COVID-19. Data were analyzed using R (version 3.6.3^37^) and RStudio (version 1.4.1100^38^). All data, scripts, and analyses are available at https://osf.io/dsvm3/.

We found no difference in risk perception depending on regional provenience, *F*(2, 71) = 0.17, *p* = 0.843, while participants differed in their perception of risk depending on the frequency with which they received information about COVID-19, *F*(3, 70) = 3.26, *p* = 0.026. Specifically, Turkey’s post-hoc tests showed that participants who indicated that they received news about COVID-19 “very often” perceived significantly more risk (*M* = 4.09; *SD* = 1.57) than participants who received the news “often” (*M* = 3; *SD* = 1.19), *p* = 0.017. We found no correlation between the number of cases and risk perception, *r*(72) = -0.021, *p* = 0.853, but found that the scores in the GAD-7 scale (measuring the general anxiety of participants^39^), were positively correlated to the perception of risks linked to COVID-19, *r*(72) = 0.33, *p* = 0.003. There was also a tendency of a positive correlation between GAD-7 scores and the frequency with which participants followed news about the COVID-19 pandemic *r*(72) = 0.20, *p* = 0.07.

### Free-listing data descriptive statistics

Free-listing data were pre-processed as follows: all punctuation characters (periods, commas, semicolons, etc.) used by participants to separate the generated words were deleted and all the words were put in separate cells. All upper-case letters were changed to lower case to allow comparison of strings. Obvious spelling mistakes and typos were corrected. Alternative spellings of the same word were unified, as well as singular and plural forms of the same word.

Participants produced a total of 169 single occurrences for COVID-19 (*M* = 4.81; *SD* = 0.80), 73% of which were produced only once by one participant. Participants produced 167 single occurrences for DISEASE (*M* = 4.81; *SD* = 0.83), with 69% of associates produced only once; 182 single occurrences for VIRUS (*M* = 4.85; SD = 0.67), with a percentage of 73% of associates produced only once; 153 single occurrences for TUMOR (*M* = 4.79; *SD* = 0.90), with a percentage of 74% of associates produced only once; 125 single occurrences for FEVER (*M* = 4.77; *SD* = 0.83), with a percentage of 74% of associates produced only once, and 144 single occurrences (*M* = 4.74; *SD* = 0.92) for FLU, with 71% of associates produced only once. Frequency distribution followed Zipf’s law^40^ typically observed in free-listing data (see also^31,41^), with fewer items produced by most participants and a long tail of less frequently produced items. As a first step, for each target concept, we identify the most frequently produced associates (listed by at least 10% of participants) and calculate their index of cognitive salience^41^. Cognitive salience is defined as the combination of two pivotal parameters in free-listing data, i.e., term frequency and its mean position, and varies between 1 and 0. Terms that are most salient for a given target concept have an index of 1, while terms that are not mentioned at all have an index of 0. Cognitive salience is thus calculated as follows: CS = F/(NmP)^41,42^, where F = term frequency, N = number of participants, and mP = mean position of the term. Once the most salient concepts associated with a target concept have been identified, we turn to a broader analysis of its conceptual representation by relying on co-occurrences—as represented by semantic networks (see e.g.^43^). Semantic networks show how salient features of each target concept are organized in the semantic space. For each target concept, we created undirected weighted semantic networks using “igraph”^44^, “ggraph”^45^, and “tidygraph”^46^ R packages. Counts of co-occurrences of bigrams (i.e., couples of words that were listed in succession) were used as direct input for constructing the networks.

### COVID-19

Table 1 shows the most frequently produced terms for COVID-19, the percentage of participants producing each term, and the cognitive salience index for each produced term.

**Table 1:** Words produced for ‘COVID-19’ by at least 10% of participants ordered according to their frequency, and their cognitive salience index.

Word	Percentage of participants producing the feature (raw frequency)	Cognitive salience
Virus	49 (36)	0.36
Pandemic	27 (20)	0.12
Fear	27 (20)	0.10
Disease	16 (12)	0.08
Death	15 (11)	0.04
Quarantine	15 (11)	0.05
Contagion	14 (10)	0.04

The first term both in terms of frequency and cognitive salience is *virus*, i.e., technically a non-superordinate concept but possibly perceived as such by laypeople. The second one for frequency (third for cognitive salience) is an emotional term, i.e., *fear*. Besides these cases, participants seem to focus mainly on the outcomes of COVID-19, both at an individual and social level (e.g., *disease*, *quarantine*). One of these possible outcomes is *death* (5th in cognitive salience).

To better visualize how participants represented COVID-19 overall, we created an undirected weighted semantic network. Words that were not listed in succession more than once were excluded from the analysis to avoid idiosyncrasies. The resulting network comprises 15 nodes (i.e., associates) and 15 edges (i.e., links). Before applying any clustering algorithms, we calculated the modularity of the network. Modularity values greater than 0 indicate non-random clustering. The modularity of COVID-19 was 0.38. We used Louvain’s algorithm for community detection to detect representative clusters of associates^47^. Communities are groups of nodes in a network that are more densely connected to one another than to other nodes. We found five different communities in our dataset. To visualize the network, we used the Fruchterman-Reingold force-directed layout algorithm.

**Figure 1:**
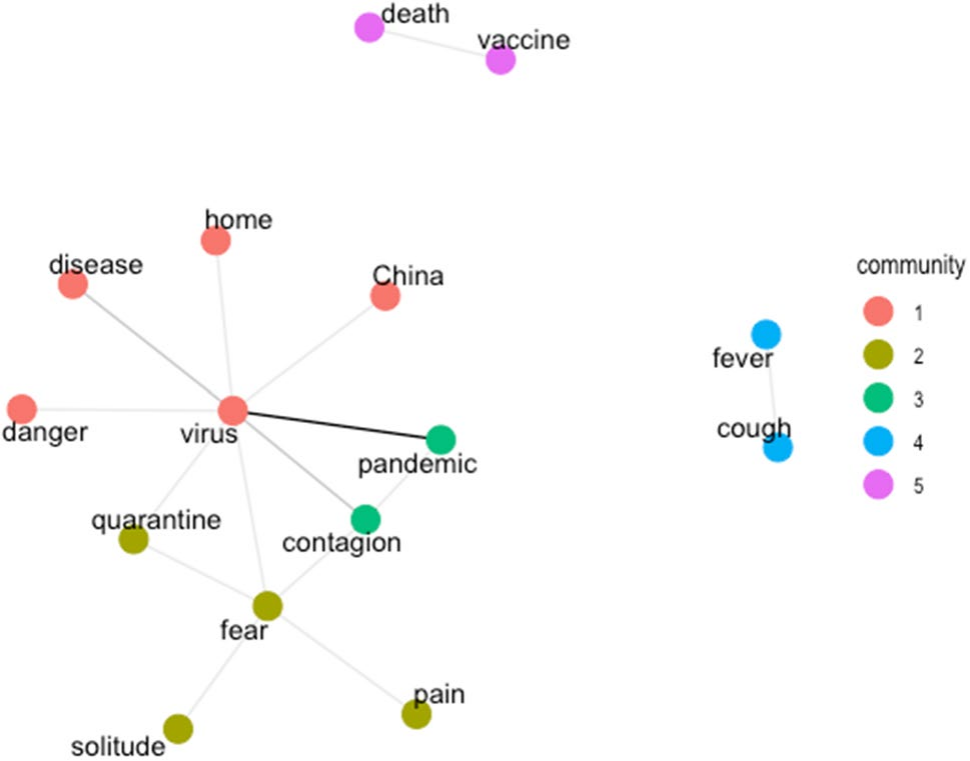
Network of words (translated in English) produced by participants in relation to *COVID-19*. Thicker links represent words that were most frequently produced together.

Figure 1 shows the semantic network for COVID-19; associates that were listed in succession (i.e., bigrams) are connected by links. Thicker links represent bigrams that were most frequently linked together. Different communities are indicated by different colors.

The term *virus* is central and associated with several other words, especially with the term *pandemic*. A clearly delimited sub-group pertains to symptoms (*cough*, *fever*), another to the spreading of COVID-19 (*contagion*, *pandemic*). The red sub-group includes more general terms (*home*, *virus*, *China*, *disease*), one of which is emotionally connoted (*danger*). Interestingly, the emotional term *fear* is not linked to *death*, but rather to *solitude*, *quarantine*, and *pain*, while *death* is linked to *vaccine*. While most terms are commonly used in other contexts not necessarily related to COVID-19, the terms *pandemic*, *China*, and *quarantine* are unequivocally linked to the novel COVID-19 emergency situation.

### Disease related concepts

The same procedure for analyzing free-listing data of COVID-19 was applied to the remaining five target concepts, all related to the semantic domain of “disease”: DISEASE*,* VIRUS*,* TUMOR*,* FEVER*,* FLU. We present these results together, focusing on differences and similarities across target concepts.

Table 2 shows the most frequently produced features (produced by at least 10% of participants) for each concept, as well as their frequency, and cognitive salience index.

**Table 2:** Words produced for ‘disease’, ‘virus’, ‘tumor’, ‘fever’, and ‘flu’ by at least 10% of participants ordered according to their frequency, and their cognitive salience index.

Target concept	Word	Percentage of participants producing the feature (raw frequency)	Cognitive salience
Disease	Cure	28 (21)	0.09
	Pain	20 (15)	0.12
	Fear	20 (15)	0.09
	Doctors	18 (13)	0.06
	Death	16 (12)	0.05
	Hospital	16 (12)	0.05
	Healing	15 (11)	0.04
	Suffering	15 (11)	0.07
Virus	Disease	30 (22)	0.14
	Contagion	22 (16)	0.12
	Fear	22 (16)	0.07
	Death	19 (14)	0.07
	Vaccine	16 (12)	0.05
Tumor	Death	39 (29)	0.14
	Disease	38 (28)	0.26
	Fear	31 (23)	0.13
	Pain	23 (17)	0.08
	Hospital	18 (13)	0.04
	Cure	16 (12)	0.05
	Chemotherapy	15 (11)	0.04
	Suffering	15 (11)	0.05
Fever	Thermometer	39 (29)	0.15
	Disease	30 (22)	0.16
	Bed	23 (17)	0.06
	Flu	16 (12)	0.07
	Fear	15 (11)	0.05
	Temperature	15 (11)	0.05
	High	14 (10)	0.14
	Medicines	14 (10)	0.04
Flu	Fever	51 (38)	0.22
	Virus	32 (24)	0.13
	Cold	28 (21)	0.13
	Disease	23 (17)	0.16
	Thermometer	16 (12)	0.06

The emotional term *fear* ranked third in cognitive salience not only for COVID-19 but also for DISEASE, VIRUS, and TUMOR, while it is fifth for FEVER and it is not produced in association with FLU. The term *death*, which is fifth in terms of frequency for COVID-19, appears in a similar position with DISEASE (5th) and VIRUS (4rth), but has a more prominent role for TUMOR (1rst). These results suggest that the concept of “COVID-19”is emotionally more similar to serious diseases and illnesses than to simple flu since it generates fear; at the same time, however, it evokes the spectrum of death less than the concept of “tumor”. In addition, VIRUS seems to be the concept semantically closer to COVID-19, with three terms in common among those most frequently produced (*fear*, *death*, and *contagion*).

To better visualize how participants represented the five disease-related concepts overall, we created undirected weighted semantic networks following the same procedures and algorithms we used for analyzing COVID-19*.* For the target concept DISEASE*,* the network is composed of 10 nodes and 10 edges (modularity = 0.36); for the target concept VIRUS the resulting network is composed of 11 nodes and 13 edges (modularity = 0.5); for TUMOR*,* the resulting network is composed of 11 nodes and 14 edges (modularity = 0.34); for FEVER, the resulting network is composed of 18 nodes and 18 edges; finally, for the target concept FLU*,* the resulting network is composed of 16 nodes and 18 edges (modularity = 0.39).

**Figure 2:**
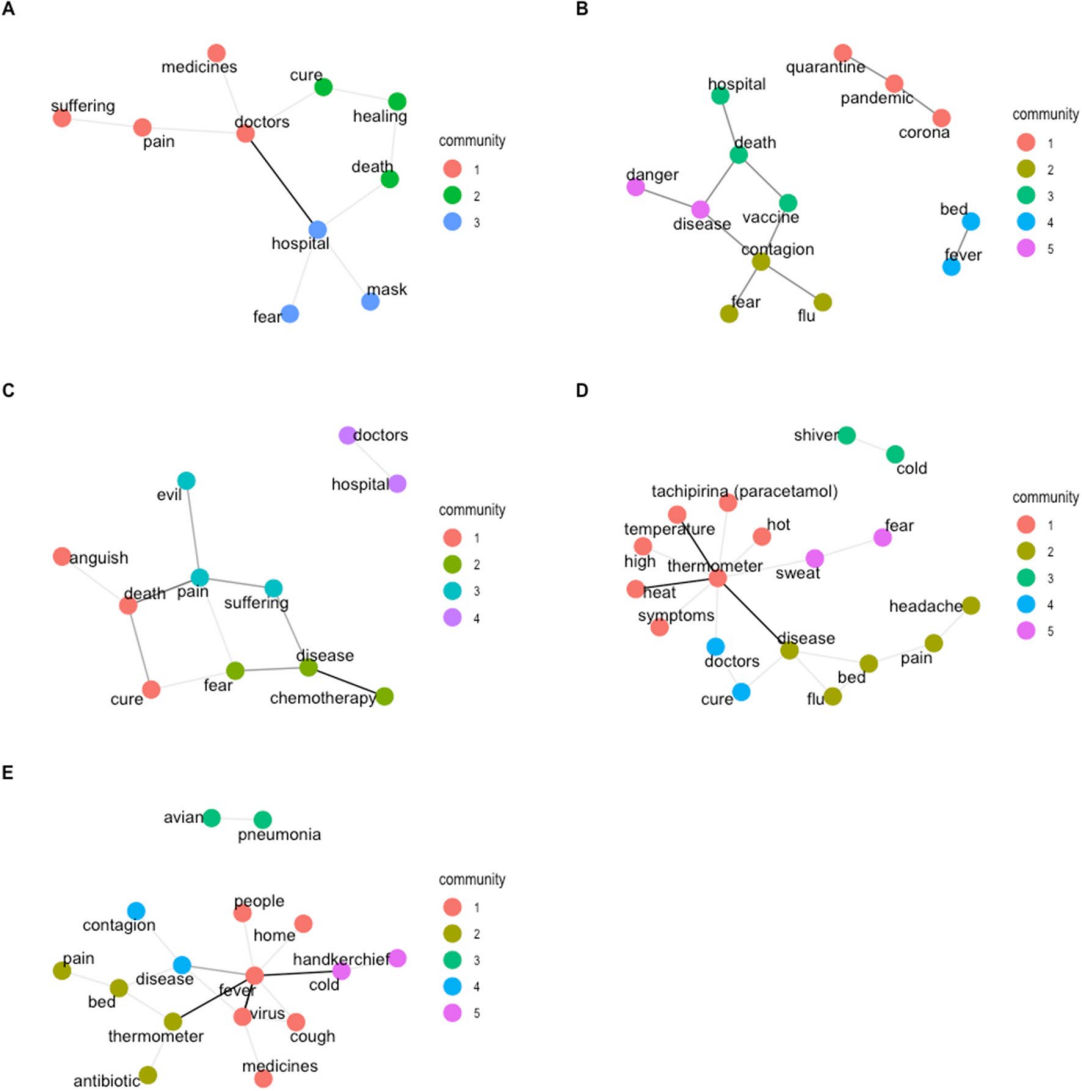
Networks of words (translated in English) produced by participants in relation to *disease* (**A**), virus (**B**), tumor (**C**), fever (**D**), and *flu* (**E**). Thicker links represent bigrams that co-occurred most frequently. Colors of communities are randomly assigned across networks, so they do not indicate similarities across concepts.

Figure 2 shows the semantic networks for DISEASE (panel A), VIRUS (panel B)*,* TUMOR (panel C)*,* FEVER (panel D)*,* and FLU (panel E); associates that were listed in succession (i.e., bigrams) are connected by links. Thicker links represent bigrams that were most frequently linked together. Different communities are indicated by different colors.

This analysis allows us to understand to what extent the current spread of COVID-19 influences the features listed for other concepts, i.e., whether the introduction of a new concept has led to a restructuring of the semantic field of other associated concepts. Although we have no data on how this semantic field was previously organized (large word association norms in Italian are not available), the production of concepts that are self-evidently COVID-19-related suggests that a change has occurred. We will therefore consider words that, within the semantic domain of each concept, refer explicitly, or unambiguously, to COVID-19.

For DISEASE, the only term unequivocally associated with the spread of COVID-19, i.e., *mask*, is linked to an emotional term, *fear*, and to *hospital*. All the terms produced in association to VIRUS are likely automatically associated with the concept of “COVID-19”. However, we can identify three interrelated words that unambiguously refer to it, i.e., *corona*, *pandemic*, and *quarantine*. Neither with the concept TUMOR nor with the concept FLU, we find terms that can be unequivocally associated with COVID-19—although we cannot exclude a possible influence. As to the concept FEVER, the two associated words *avian*–*pneumonia*, even if not unambiguously referred to COVID-19, are very likely influenced by its spread. Below we will briefly discuss highlights from the networks resulting from participants’ responses to the disease-related target concepts.

In the network resulting from the free listing of DISEASE, we found three main communities. One mainly related to the role of doctors, and overall negatively connotated (*suffering, pain, doctors, medicines*); the second one contains possible outcomes of being ill (*cure, healing, death*). The last community is the one more probably affected by COVID-19-related experiences (*hospital, mask, fear*).

The network composed of associates to VIRUS resulted in five communities. Two among these stand alone: one explicitly related to COVID-19 (*quarantine, pandemic, corona*), and the second one composed only of *fever* and *bed.*

The network of TUMOR appears as the most emotionally loaded. In fact, out of four communities, only one does not contain explicit emotional terms (*doctors, hospital*, which is an isolated community in the network).

In the FEVER network, communities refer mostly to symptoms; interestingly, the influence of the current situation related to COVID-19 might be reflected by the presence in the network of the term *fear*.

The last network, FLU, is composed of five communities, with one of these that stands alone (*avian, pneumonia*, possibly an association with other virus outbreaks triggered by the COVID-19 emergency).

### COVID-19 and disease-related concepts shared semantic space

Among all the associates produced for all five disease-related concepts and COVID-19*,* we found seven common words: *fear, danger, anxiety, cure, doctors, medicines,* and *hospital*. Table 3 shows the frequencies of production of each of the seven common words across the six concepts.

**Table 3:** Words in common among the six target concepts and percentage of participants producing the word for each concept. Raw frequencies are given in brackets.

Common words	COVID-19	Disease	Virus	Tumor	Fever	Flu
Fear	27 (20)	20 (15)	22 (16)	31 (23)	15 (11)	7 (5)
Danger	7 (5)	3 (2)	7 (5)	3 (2)	5 (4)	3 (2)
Anxiety	4 (3)	7 (5)	3 (2)	1 (1)	1 (1)	1 (1)
Cure	3 (2)	28 (21)	9 (7)	16 (12)	8 (6)	7 (5)
Doctors	1 (1)	18 (13)	3 (2)	5 (4)	9 (7)	9 (7)
Medicines	1 (1)	11 (8)	5 (4)	3 (2)	14 (10)	12 (9)
Hospital	1 (1)	16 (12)	7 (5)	18 (13)	4 (3)	1 (1)

Looking at the percentages, COVID-19 is the only concept, together with VIRUS, for which the term *fear* alone is highly frequent, followed by two further emotional terms, *danger* and *anxiety*, which are in any case much less frequent. Compared to other concepts, COVID-19 evokes fewer possibilities of cure and healing, probably because it is less known. It has to be borne in mind that these data were collected during the first COVID-19 outbreak, when the possibility of a vaccine was still far from its current development. For example, with the concept TUMOR, the word *fear* is also the most frequent word, but also *cure* and *hospital* are highly frequent. With the concept DISEASE, and even more with FEVER and FLU, the focus is not only on fear but on the concrete possibilities to deal with the illness: in fact, *hospital*, *medicines*, *doctors* are highly frequent words.

To further investigate commonalities across concepts, we inspected the ANEW database^48^ searching for the seven terms shared across the six target concepts. ANEW provides affective norms for over 1000 Italian words, measuring on 9-point Likert scales Valence (1 = very unpleasant; 9 = very pleasant); Arousal (1 = very calm; 9 = very aroused); Dominance (1 = very submissive; 9 = very dominant) and Concreteness (1 = abstract; 9 = concrete), among other psycholinguistic variables. We found 4 of the seven words in the database, i.e., *fear,* valence *M* = 2.21 (*SD* = 1.45); arousal *M* = 6.94 (*SD* = 2.25); dominance *M* = 3.52 (*SD* = 2.08); concreteness *M* = 5.00 (*SD* = 2.96), *medicine,* valence *M* = 4.93 (*SD* = 2.47); arousal *M* = 5.59 (*SD* = 2.09); dominance *M* = 4.41 (*SD* = 2.07); concreteness *M* = 6.30 (*SD* = 1.87), *hospital,* valence *M* = 2.83 (*SD* = 1.84); arousal *M* = 6.71 (*SD* = 1.82); dominance *M* = 4.06 (*SD* = 2.07); concreteness *M* = 8.45 (*SD* = 0.76) and *danger*, valence = 2.28 (*SD* = 1.59); arousal = 7.25 (*SD* = 2.18); dominance = 4.16 (*SD* = 2.23); concreteness *M* = 5.35 (*SD* = 2.32). We also found *anxious* (instead of anxiety), valence *M* = 2.16 (*SD* = 1.22); arousal *M* = 7.25 (*SD* = 1.77); dominance *M* = 3.16 (*SD* = 2.01); concreteness *M* = 4.90 (*SD* = 2.38). Overall, it seems the six target concepts share an intense emotional load, mainly related to sensation of instability and arousal (for similar findings on Italian emotional responses to COVID-19 emergency see e.g.^49^).

#### Correspondence analysis

**Figure 3:**
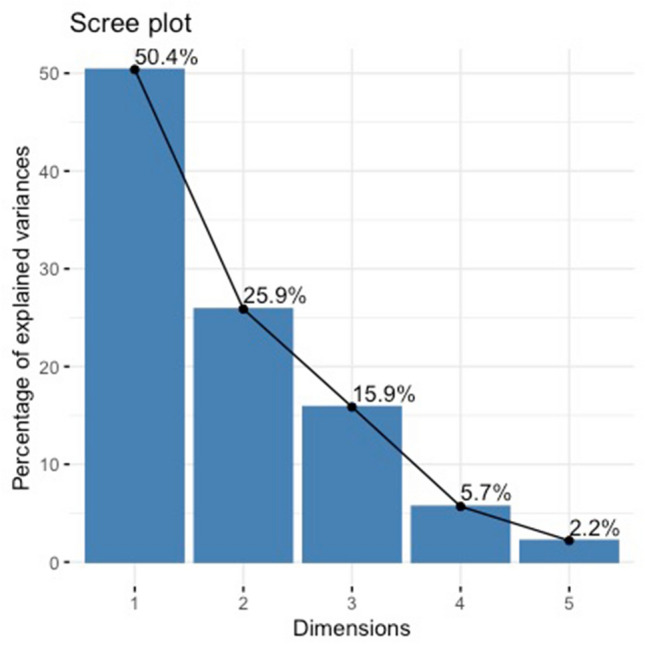
Percentage of variance explained by dimensions extracted by correspondence analysis.

To further assess the semantic similarity and diversity across the six concepts, we conducted a correspondence analysis, implemented through the “FactoMineR” and “factoextra” R’s packages^50,51^. Correspondence Analysis is a data reduction technique allowing to extract the main dimensions along which semantic information is grouped (^52^, see also^53,54^). The input for the following correspondence analysis is a matrix constructed relying on words that were produced by at least 10% of participants for COVID-19 (rows) and their frequencies across all six concepts (columns). The identified words were differentially distributed across the six concepts, ${\rm X}^{2}$(30) = 230.65, *p* = 0.002. We found the first two dimensions explained 76% of the variance, with Dimension 1 explaining most of the variance (50.38%), followed by Dimension 2 (25.87%); this can be seen in Fig. 3.

**Figure 4:**
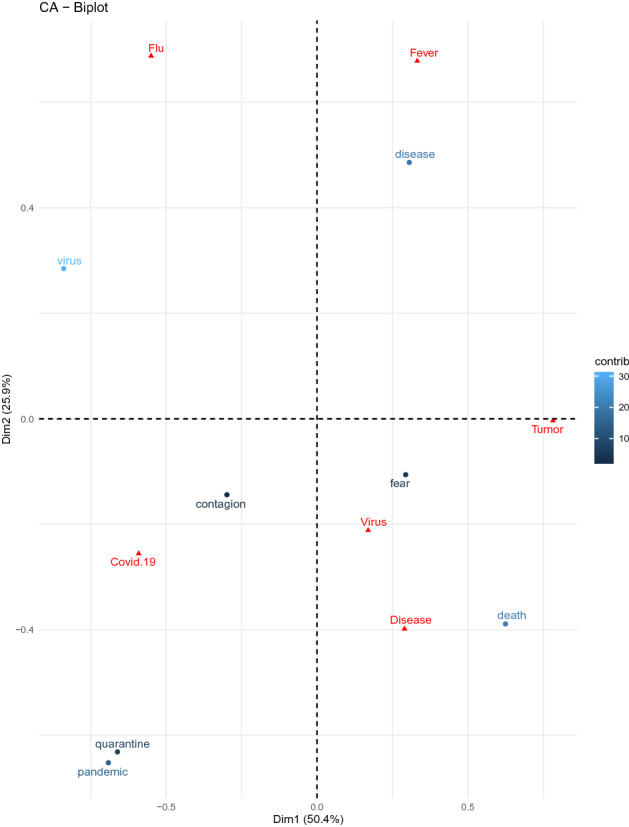
Plot of dimensions 1 and 2 of words that were most frequently produced for *COVID-19,* and their relation with the target concepts.

The results of the correspondence analysis allow us to clearly understand the particularity of the pattern of features elicited by COVID-19 with respect to those evoked by the other target concepts. Figure 4 shows the results of the correspondence analysis. Target-concepts are represented in red, words that were produced in association with COVID-19 are represented in blue. Lighter blue indicates the word has a stronger contribution to the Dimensions.

We discuss only concepts and features weighing more than 10% of the variance in each dimension. On the first Dimension (50.4% of the overall variance) COVID-19 and FLU (even if FLU insists more on Dimension 2) are opposed to TUMOR. COVID-19 and FLU are associated with *virus* and *pandemic* (even if the term *pandemic* has a higher weight on Dimension 2), while TUMOR is associated with *death*. On the second Dimension (25.9% of the overall variance) FLU and FEVER are associated with the term *disease* and opposed to COVID-19 (even if the concept insists more on Dimension 1) and to the words *pandemic*, *quarantine*, and *death* (even if the term *death* has higher weight on Dimension 1). The interpretation of the correspondence analysis evidences that participants represent ‘COVID-19’ as differing from a severe disease like ‘tumor’, because less mortal and more contagious. At the same time, they do not seem to assimilate it to a normal ‘flu’ or to reduce it to the symptom of ‘fever’, since it is more contagious (*pandemic*), it has practical and impacting consequences (*quarantine*), and it can eventually be lethal (*death*).

## Discussion

In the present study, we were interested in assessing the conceptual structure of a newly introduced–and yet extremely salient–concept and its possible impact on similar semantic domains. To this end, we tested how Italian participants during the first lockdown represented the concept COVID-19, which features it elicited, and its similarity and differences with less novel concepts referring to the same semantic domain—that of (more or less severe) diseases, using a free-listing task.

Overall, the results offer a very clear pattern. First, our sample’s representation of COVID-19 is strongly emotionally connoted. *Fear* is the most frequently produced term, after the term *virus*. Compared to other disease-related concepts, COVID-19 is the only concept that is highly associated with a single term, an emotional one, i.e., *fear*. *Fear* (27%) is produced far more frequently than all other features. Interestingly, it is followed at a distance by two further emotional terms, *danger* and *anxiety*. This is in line with preliminary results^49^ that showed through an analysis of linguistic networks of Italian hashtags performed during the initial phase of lockdown an intense and complex emotional pattern related to COVID-19 pandemics. We can better qualify this result by noticing that, differently from all other diseases, COVID-19 hardly evokes terms related to hospitals, cures, doctors, medicines. Aside from *fear* and *death*, the features produced are either superordinate terms (*virus*, *illness*), or terms related to the new phenomenon (*pandemic*, *contagion*, *quarantine*). TUMOR also evokes *fear*, but it also yields the words *hospitals* and *cures*. This is not the case for COVID-19, where *fear* is likely associated with its mysterious nature, and especially to the fact that little is known as to how to deal with it. Interestingly, the network analysis suggests that *fear* is also related to scary scenarios owing to changes in social relations (*solitude*, *quarantine*). The emotional activation is likely linked mostly to its vagary and indeterminacy, to the feeling of not knowing how to deal with it, and to its social consequences, rather than to its strong association with *death*—differently from what happens with TUMOR.

Second, we found evidence suggesting a possible modification of the semantic field of diseases, as a consequence of the introduction of a novel concept such as COVID-19. Although we have no data collected before the spread of Covid-19 to perform a comparison, the correspondence analysis shows that COVID-19 has an important weight on both Dimension 1 and 2. The introduction of COVID-19 seems to modify the distinction between severe diseases, such as TUMOR, and mild ones, such as FLU. COVID-19 is conceived as less mortal than TUMOR but as more mortal than FLU. Compared to TUMOR, but also to FEVER and FLU, people represent COVID-19 as more contagious and as endowed with significant social consequences, such as *quarantine*.

Third, the emergence of the concept of COVID-19, led not only to change the semantic space of diseases but also to the restructuring of patterns of conceptual relations activated by associated concepts, such as those of DISEASE, VIRUS, and FEVER. Specifically, when asked to list their features, participants produced features explicitly and unequivocally elicited by COVID-19, such as *mask* and *pneumonia*. Such modifications are less present, or less clearly apparent, for concepts characterized by a more established and stable representation, like TUMOR and FLU, even if the association with COVID-19 is likely to have triggered the feature *fear*, produced with FLU.

Even if the generalizability of our results is limited by the small-scale focus on the Italian sample in a specific timeframe, these findings provide an initial and illuminating picture of the perception of COVID-19 during lockdown. Our study clearly indicates the need for large-scale cross-cultural and longitudinal studies. In fact, another way to approach this topic would be analyzing natural language use (e.g., from social media^49^, or^55^), as it has been done for other countries^56^. Other studies concerned with the perception of COVID-19 in the Italian lockdown have focused on large corpora derived from social media discourses (e.g.^49,57^). However, to date our study is the first exploring this issue using a semantic fluency task, that while not detecting natural language use has the advantage of explicitly tapping into participants’ perceptions.

## Conclusions

Overall, our results have implications for policies on COVID-19. Knowing how people represented it during the first wave can help politicians and scientists to operate during the possible following waves. Furthermore, they have wide implications for studies on concepts, and more specifically for research on conceptual flexibility. The pattern of associated features shows that we mainly represent the concept of COVID-19 in terms of one dominant emotion, i.e., *fear*. The correspondence analysis on COVID-19 and other associated concepts suggests that the introduction of this new concept led to restructuring the semantic field of diseases, as it is represented as contrasting both to mild and severe diseases. Finally, the pattern of features produced with the associated concepts reflects the influence of the pandemic situation in which participants were.

To conclude, we showed how people during the first Italian lockdown represented COVID-19, and how they understood it compared to other concepts in the ‘disease’ domain. The use of a free-listing method allowed us to tackle people’s perceptions directly. Our results highlight how rich a novel concept can be and even suggest that introducing a novel concept might rapidly modify previous knowledge, allowing us to appreciate the exquisite flexibility of our concepts.

## Methods

### Participants

Ethics permission was granted by the Ethics Committee of the Department of Dynamic and Clinical Psychology, Sapienza University of Rome (Prot. no. 000275—23/03/2020). All methods conformed to the Declaration of Helsinki. Before completing the survey, participants were informed of the general purpose of the study and provided informed consent.

A total of 74 Italian participants took part in the study in a window of time between April, 2nd and May, 14th 2020—i.e., in the initial phase of the first Italian lockdown. The questionnaire was implemented in Qualtrics. Participants were contacted via anonymous link either by posting the questionnaire on social networks (Facebook, Twitter) or spreading the questionnaire through the research team’s extended network of acquaintances. Originally we contacted 166 people, but 90 of them did not complete the questionnaire, likely because it required a long time (see below) and we allowed participants to interrupt and continue it later within a 3-days-time (*n* = 88). A small percentage of participants (*n* = 2, 1.2%) instead completed the task, but typed answers not congruent with what we were asking (e.g., swear words, symbols), so their responses were not considered for the analyses. From the remaining 76 participants, we excluded data from participants who indicated that their nationality was other than Italian (*n* = 2, 2.63% of the sample), as we were specifically interested in testing people sharing a common cultural milieu. The final sample is therefore composed of 74 participants (50 females, *M* age = 37.46; *SD* = 12.46; 24 males, *M* age = 42.20; *SD* = 14.64). All socio-demographic information collected is reported in Appendix A (see Supplementary Materials).

### Design and procedure

Participants took part in an online survey, divided into three sections. They completed the three sections in a fixed order. In the first part of the survey, participants completed the free-listing task. In the second part of the survey, participants were asked to complete four scales: the Interpersonal Reactivity Index (IRI)^58–60^, testing their general empathy; the Multidimensional Assessment of Interoceptive Awareness (MAIA)^61^ investigating their interoceptive awareness; the Stereotype Content Model (SCM)^62^, that investigates the content of the stereotypes endorsed by individuals towards specific social groups; and the Generalised Anxiety Disorder-7 (GAD-7)^39^ aimed at measuring the severity of Generalized Anxiety Disorder. Here we will focus only on GAD-7, because it is directly relevant to the purposes of this study. In the third part of the survey, we collected socio-demographic information. We asked participants to report their age, birth sex, level of education, profession, birth nation, city and region of provenience, current health condition, current way of living (confined or not confined to the house), frequency with which participants received information and news about COVID-19, and personal perceived risk to contract COVID-19 (see Appendix A, Supplementary Materials).

In the first part of the survey, containing the free-listing task, participants were asked to list the first five words that came to their mind in relation to the words presented. We encouraged them to respond as quickly as possible, without spending too much time thinking about every single word. Participants typed their responses into separate text boxes for each target word.

The free-listing section was designed as follows: participants responded to a total of 96 target words, divided in 11 categories (e.g., emotional words, words referring to the body, to the family, institutional words) (see Appendix B, Supplementary Materials), and randomly presented. To better distinguish target concepts and associated features, throughout the paper we will refer to the first ones with upper case letters, while the second ones will be given in italics. For the present study and analyses, we took into account only the six words of the “disease” category: COVID-19*,* DISEASE*,* VIRUS*,* TUMOR*,* FEVER*,* and FLU. To select words of the “disease” domain we used the word COVID-19, with two superordinate terms of different level of generality (VIRUS, DISEASE), two coordinate concepts referring to more or less severe diseases (FLU, TUMOR) and a term referring to a symptom that characterizes a variety of diseases (FEVER).

## Supplementary Information

Supplementary Information.

## Data Availability

All data and scripts are available at https://osf.io/dsvm3/.
