# Supplementary material for: Differences and similarities in the conceptualization of COVID-19 and other diseases in the first Italian lockdown
Source: Sci Rep. 2021 Sep 15;11:18303. doi: 10.1038/s41598-021-97805-3 (PMC8443562; doi:10.1038/s41598-021-97805-3)
Supplement: Supplementary file 1 — Supplementary Information. [file 41598_2021_97805_MOESM1_ESM.pdf]

Differences and similarities in the conceptualization of COVID-19 and other diseases in the first Italian lockdown

Claudia Mazzuca[1], Ilenia Falcinelli[1°], Arthur-Henri Michalland[2°], Luca Tummolini[3], Anna M. Borghi[1,3]

[1]Department of Dynamic and Clinical Psychology, Sapienza University of Rome, Rome, Italy
[2] University of Montpellier - LIFAM, Montpellier, France
[3] Institute of Cognitive Sciences and Technologies, Italian National Research Council, Rome, Italy

Supplementary materials

Appendix A. *Sociodemographic Information of Participants*

Sociodemographic Information	Full sample	
	n	%
Title of study		
Junior High School	1	1
High School	17	23
Bachelor's/Master's degree	30	41
PhD/Post-lauream qualification	26	35
Profession		
Student	18	24
Unemployed	6	8
Retired	3	4
Employed	47	64
Modality of working during Covid-19 emergency		
Smartworking	35	74
Not working	8	17
In workplace	4	9

Dismissal caused by Covid-19 emergency		
Yes	0	0
No	47	100
Part of Italy		
North	22	30
Centre	37	50
South	15	20
Current health condition		
Healthy	73	99
Positive swab to Covid-19	0	0
Negative swab to Covid-19	0	0
Sick with other pathologies	1	1
Current way of living		
Confined to house	64	86
Not confined to house	10	14

Note. $N = 74$. Participants were on average 39 years old ($SD = 13.31$)

Appendix B. *Categories and Target Words of the Free-Listing Task*

Category	***Target Words***
Diseases	*Covid-19, Disease, Virus, Tumor, Fever, Flu*
Epidemiological Concepts	*Pandemic, Transmission Rate, Exponential Growth, Herd Immunity, Social Distancing*
Philosophical-Religious Concepts	*Life, Religion, Faith, Future, Prayer, Salvation, Mourning, Present, Responsibility, Destiny, Death, Decease*
Emotional Concepts	*Love, Affection, Altruism, Optimism, Trust, Hope, Fear, Anxiety, Uncertainty, Panic, Threat, Danger*
Concepts referred to Body and Senses	*Body, Corpse, Hand, Mouth, Eyes, Face, Blood, Breath, Vision, Touch, Head, Stomach*
Social Professions	*Scientist, Doctor, Nurse, Teacher, Journalist, Prime Minister, Priest*
Social-Collective Concepts	*Crowd, People, Demonstration, Parade, Muster, Huddle*
Concepts referred to Family	*Family, Partner, Brother, Sister, Mother, Father, Wife, Husband, Son, Grandparent, Children, Elderly*
Institutional Concepts	*Government, State, School, University, Democracy, Internet, Nation, Science, Civilization, Money, Morality, Norm*
Places-Means of Transport	*Hospital, House, Airport, Station, Boat, Car, Border*
Nations	*Italy, France, England, United States, China, Europe*
